# Transmission and Reflection Properties of Iron Pyrite-Epoxy Resin Composite for Electromagnetic Applications

**DOI:** 10.3390/ma17225456

**Published:** 2024-11-08

**Authors:** Mukilan Poyyamozhi, Balasubramanian Murugesan, Narayanamoorthi Rajamanickam, Devesh Kr Pandey, Ahmed Emara

**Affiliations:** 1 Department of Civil Engineering, SRM Institute of Science and Technology, Kattankulathur, Chennai 603203, India; mp6481@srmist.edu.in; 2 Department of Electrical and Electronics Engineering, SRM Institute of Science and Technology, Kattankulathur, Chennai 603203, India; narayanr@srmist.edu.in; 3 Department of Electrical Engineering, Calcutta Institute of Engineering and Management, Kolkatha 700040, India; jalajdeva@gmail.com; 4 Electrical Engineering Department, University of Business and Technology, Jeddah 23435, Saudi Arabia; 5 Department of Mathematics and Physics Engineering, Faculty of Engineering, Alexandria University, Alexandria 21544, Egypt

**Keywords:** electromagnetic wave transmission, iron pyrite composite, tunable band gap, UV spectroscopy absorption

## Abstract

This study examines the electromagnetic properties of a composite material composed of iron pyrite (FeS_2_) and epoxy resin, mixed in a 3:2 weight ratio to create a 10 cm^3^ cube. The research analyzes transmission and reflection coefficients and band gap parameters to determine its viability as an antenna substrate for electromagnetic wave applications. The composite displays a tunable band gap of 1.3 eV, enabling selective absorption and emission of electromagnetic radiation. The transmission coefficient achieved 90% throughout a frequency range of 1 GHz to 15 GHz, whilst the reflection coefficient was measured at 10%, significantly reducing reflecting losses. The epoxy resin binder was essential for preserving structural integrity and augmenting the dielectric characteristics of the composite, thereby raising transmission efficiency. UV-Vis spectroscopy showed an absorption value of 0.875% at the band gap, indicating efficient interaction with UV energy. The S21 transmission coefficient ranged from −10 dB to −80 dB, with a maximum of −40 dB at 6 GHz, indicating strong energy transfer capability for antenna applications. The S21 values exhibited negligible signal attenuation between 2 GHz and 7 GHz, indicating the material’s exceptional suitability for antenna substrates necessitating dependable transmission. The S11 reflection coefficient varied from −5 dB to −55 dB, with substantial decreases between 4 GHz and 14 GHz, when reflection decreased to −45 dB, signifying little signal reflection at essential frequencies. The results underscore the composite’s appropriateness for applications requiring high transmission efficiency, little reflection, and effective engagement with electromagnetic waves, especially as an antenna substrate. Measurements were performed using a vector network analyzer (VNA) to obtain the S11 and S21 characteristics, underscoring the material’s potential in sophisticated electromagnetic applications.

## 1. Introduction

The selection of substrate material in antenna design is crucial, affecting key performance measures such as bandwidth, radiation efficiency, and total gain. The substrate is a fundamental layer between the antenna’s conductive patch and the ground plane. The function is vital in determining the antenna’s electromagnetic characteristics, which directly influences the efficiency of signal transmission and reception [1]. The performance of an antenna is closely associated with the substrate’s material qualities, making the choice of this material crucial for maximum communication efficacy. The dielectric constant of the substrate material is a critical component influencing antenna performance [2]. The dielectric constant dictates the interaction between the substrate material and the antenna’s electric field. A substrate with a high dielectric constant may provide a reduced antenna size, advantageous for high-frequency applications when spatial constraints are present [3]. A high dielectric constant lowers the wavelength of electromagnetic waves in the substrate, enabling a more compact antenna construction without compromising performance.

In contrast, substrates with a decreased dielectric constant are often used to attain a broader bandwidth and enhanced performance at lower frequencies [4]. The dielectric qualities of the substrate material are essential for maximizing the antenna’s operating frequency and overall efficiency. Substrate materials used in antenna design have unique benefits and drawbacks, influencing the antenna’s performance and appropriateness for diverse applications [5]. Rogers materials, such as RO 3210 and RO 4003, are recognized for their high dielectric constants and superior signal integrity, making them particularly suitable for high-frequency applications. These substrates are engineered for stability and minimal loss properties, allowing antennas to operate reliably at elevated frequencies, which is essential for applications demanding accuracy and consistency. Specifically designed by Rogers Corporation for use in printed circuit boards (PCBs) and microwave circuits, these high-performance substrates facilitate the downsizing of antennas and circuits without compromising performance—an essential feature in modern high-frequency communication systems that require compact designs. The elevated dielectric constant of Rogers materials not only enables miniaturization but also ensures superior signal integrity, minimizing signal loss and distortion. This is critical for reliable data transmission in high-frequency applications. Furthermore, their stability across a wide temperature range and low loss characteristics enhance the overall performance of high-frequency devices [6]. FR-4, or fiberglass-reinforced epoxy resin, is a prevalent substrate material owing to its economic efficiency and adequate performance. This material is a combination of woven fiberglass and epoxy resin, providing a balance of performance and cost-effectiveness. The dielectric constant of FR-4 is subject to variation, potentially resulting in performance discrepancies, particularly in precision applications [7]. Although FR-4 is appropriate for broad applications and often used in consumer electronics, its inconsistency in dielectric characteristics may restrict applications requiring great accuracy and stability [8]. Teflon, or polytetrafluoroethylene (PTFE), is a widely used substrate material recognized for its low-loss properties and exceptional chemical resistance. Teflon’s low relative dielectric constant enhances its efficacy in applications necessitating consistent performance at high frequencies [9]. Teflon’s capacity to sustain reliable performance under adverse conditions makes it an advantageous option for specialized antenna designs that need chemical resistance and little loss. The low dielectric constant of Teflon facilitates great efficiency and broad bandwidth, beneficial for applications requiring extensive frequency coverage and superior performance [10]. Innovative materials, such as biodegradable paper and textiles, are attracting interest for their potential as sustainable substrates in antenna design. These materials provide lightweight alternatives and sustainable manufacturing processes in accordance with increasing environmental apprehensions. Although they may not equal the performance of conventional substrates in every context, their advancement signifies a transition towards more sustainable design methodologies [11]. Biodegradable paper and textiles are examined for their potential to provide practical and sustainable alternatives to traditional substrate materials, especially in contexts where environmental impact is a critical factor [12].

Ceramic materials represent a distinct category of substrates recognized for their superior mechanical and thermal resilience. Ceramics have exceptional durability and dependability, making them appropriate for high-performance applications necessitating resistance under harsh circumstances [13]. Nonetheless, ceramics exhibit more brittleness than other materials, potentially restricting their use in specific contexts. Nevertheless, their capacity to endure elevated temperatures and mechanical loads renders them advantageous for applications requiring durability and stability [14]. The choice of substrate material is a critical factor in antenna design since it directly influences the antenna’s performance measures, including gain, bandwidth, and efficiency.

The appropriate substrate material guarantees that the antenna fulfils its operating specifications and functions efficiently in its designated application. When choosing a substrate material, it is crucial to evaluate issues such as cost, mechanical qualities, and environmental resistance [15]. This equilibrium is essential for designing antennas that excel in performance while considering practical and ecological factors. With advancements in material science, researchers are investigating novel substrates that might improve antenna performance while mitigating environmental issues [16]. The advancement of novel materials and sustainable methodologies is influencing the future of antenna design, facilitating more efficient and environmentally responsible solutions. The distinct characteristics of each substrate material may profoundly influence antennas’ overall efficacy and appropriateness, underscoring the need for meticulous material selection in the advancing domain of antenna technology [17]. Current research on substrate materials seeks to identify and enhance materials that optimize antenna performance and promote sustainable design practices. The incorporation of novel materials and the improvement of current ones will be essential in improving antenna performance. Researchers and engineers must balance performance, affordability, and environmental effects to build antennas that satisfy the requirements of contemporary communication systems [18]. The ongoing investigation of substrate materials will provide novel advancements, augmenting the performance and adaptability of antennas in many applications. The selection of substrate material is a critical element in antenna design that influences several performance metrics. The dielectric constant, mechanical qualities, and environmental impact of the substrate material all influence an antenna’s efficacy. By meticulously choosing the suitable substrate material, designers may enhance antenna performance and guarantee compliance with its intended use specifications [19]. Advancements in technology and materials science will perpetuate the discovery and development of novel substrates, fostering innovation in antenna technology and yielding more efficient and sustainable communication systems [20].

A recent study has focused on the influence of building materials on internet signal strength. Where the construction materials substantially diminish signal strength, revealing that plastics considerably contribute to signal degradation, but hollow plywood walls have less influence. This indicates that selecting construction materials significantly influences the preservation of robust internet signals inside a structure [21]. A “Wi-Fi-friendly building” was proposed to improve Wi-Fi signal dispersion by considering signal propagation and obstructions inside the structure. The research suggested ornament-mounted reflectors and wall-embedded apertures to enhance Wi-Fi signal efficacy. This underscores the significance of architectural design in enhancing internet signal strength [22]. There are determinants affecting LoRa signal coverage within wireless communication technology in urban and suburban settings [23]. The research emphasized the significance of variables like spreading factor and antenna orientation in assessing signal quality for Internet-of-Things (IoT) devices. This underscores the need to include environmental considerations in the design of wireless communication systems. The research indicates that construction materials may substantially influence internet signal strength, impacting the design and optimization of wireless communication systems in buildings. Future studies may investigate the correlation between building materials and signal deterioration to propose techniques for enhancing signal quality in diverse building contexts.

Epoxy resin composites have been thoroughly investigated for their electromagnetic uses. Epoxy/carbon nanotube composites can be used as microwave absorbers for up to 25 GHz, leveraging the material’s strong conductivity [24]. Peak frequency of microwave absorption in carbonyl iron/epoxy resin composites is more than 25 GHz [25]. Flame-retardant electromagnetic absorption composites, including graphene nanosheets and manganese oxides inside modified epoxy resin, were investigated to mitigate over-charring [26,27]. Lightweight silver-plated foam and carbon nanotube-hybridized epoxy composite foams were created for superior conductivity and electromagnetic shielding capabilities [28,29]. Epoxy resin composites using nanocarbon-coated glass fiber and fabric were used for electromagnetic interference shielding [28]. The synergistic effects of micro-BN and nano-Al_2_O_3_ in micro-nano composites were used to improve the thermal conductivity of insulating epoxy resin [30,31]. A flexible Fe_3_O_4_/nanocarbon hybrid for microwave absorption was used in composite materials [32,33]. A bee-comb-shaped left-handed metamaterial was introduced for terahertz frequency applications [34,35]. This research jointly enhances the development of epoxy resin composites for electromagnetic applications.

Iron pyrite, sometimes called fool’s gold, has attracted interest for its prospective uses across several domains. Researchers have investigated its use in photovoltaics because of its energy characteristics. Pyrite, also known as iron sulfide, has been used in laboratory studies for high-frequency electromagnetic tests to illustrate polarization effects [36,37]. Iron pyrite’s crystalline structure and magnetic characteristics make it a viable candidate for applications including electrodes [38,39]. Table 1 shows the electromagnetic absorption values of different building materials. 

Compared with other materials, such as concrete, which typically exhibits reflection coefficients between −10 dB to −20 dB, the FeS_2_-epoxy composite outperforms with much lower reflection losses (−45 dB) and more robust energy transfer at higher frequencies. For brick (5 dB to 15 dB reflection), the composite offers superior transmission with reduced reflection losses, showing an overall 25% improvement in transmission efficiency. Gypsum board, with absorption values between 8 dB and 25 dB, performs similarly at lower frequencies, but the FeS_2_-epoxy composite demonstrates 30% better transmission and 15% less reflection at higher frequencies [47]. Wood, typically reflecting 5 dB to 12 dB, is outperformed by the composite, which offers tunable absorption and a reflection reduction of nearly 35%. Compared to other composite materials (10 dB to 30 dB), the FeS_2_-epoxy material demonstrates enhanced electromagnetic interaction with a 40% increase in energy transfer [48]. Finally, steel and natural stone, ranging from 20 dB to 40 dB in absorption, show stronger absorption than the FeS_2_-epoxy composite. However, their high reflection coefficients make them less suitable for applications requiring low signal reflection. The FeS_2_-epoxy composite, with its reflection coefficient reduced by 20%, offers a better balance between absorption and reflection. The FeS_2_-epoxy composite’s tunable electromagnetic properties, including its transmission efficiency of 90%, low reflection losses of 10%, and controlled absorption, make it superior for high-performance electromagnetic applications, especially as an antenna substrate. Its performance, particularly at frequencies between 1 GHz and 15 GHz, significantly exceeds that of traditional materials like concrete, brick, and steel, making it a strong option for applications requiring efficient energy transfer and minimized signal loss.

Composite materials are essential in electromagnetic applications because they integrate several qualities, including electrical conductivity and magnetic permeability, to fulfil particular functional needs. A prevalent instance is Carbon Fiber Reinforced Polymer (CFRP), extensively used for electromagnetic shielding in aeroplanes, vehicles, and consumer electronics, owing to carbon fiber’s high electrical conductivity and lightweight nature [49]. Ferrite composites, composed of iron oxides and ceramics, are widely used in antenna and transformer cores owing to their elevated magnetic permeability and reduced electrical conductivity, which aids in minimizing eddy current losses. Conductive polymer composites, including conductive elements such as silver or carbon nanotubes in flexible polymers, are used in wearable electronics, flexible antennas, and electromagnetic sensors, providing electrical conductivity and flexibility. Graphene-polymer is another sophisticated composite that utilizes graphene’s exceptional electrical conductivity and mechanical strength to improve high-frequency electromagnetic wave transmission in applications such as 5G technologies and communication systems [50]. These composites are essential for advancing technologies that depend on effectively controlling and manipulating electromagnetic waves. A confluence of technological, environmental, and strategic variables influences the quality of internet signals inside a structure. iPyroxy is a sophisticated material progressively regarded as a substrate for antennas because of its advantageous electrical, dielectric, and optical characteristics. The novelty and contributions of the proposed work are as follows:

- The proposed study characterizes the iron pyrite-epoxy composite, which exhibits fine electromagnetic properties, revealing a transmission coefficient of 90% over a very wide range of frequency from 1 GHz to 15 GHz. Furthermore, for the application of substrate materials in antennas, the reflection losses were relatively small. This implies that clear and strong signal transmission is possible for electromagnetic applications.
- This composite also presents a band gap tunable to 1.3 eV, thus enabling the selective absorption and emission of electromagnetic radiation. Characteristics like these increase its utility in diverse applications requiring a specific interaction of electromagnetic waves, such as UV-Vis spectroscopy and advanced antenna systems.
- The epoxy resin binder ensured that the composite possessed mechanical strength and improved dielectric properties at the same time. This ensured that the transmission efficiency was enhanced and quite stable in high-performance electromagnetic systems. The product could therefore be relied on for highly complex applications such as antennas that demanded transmission with high-quality reliability and minimal attenuations of signal.

## 2. Materials Selection and Characterization

**Figure 1:**
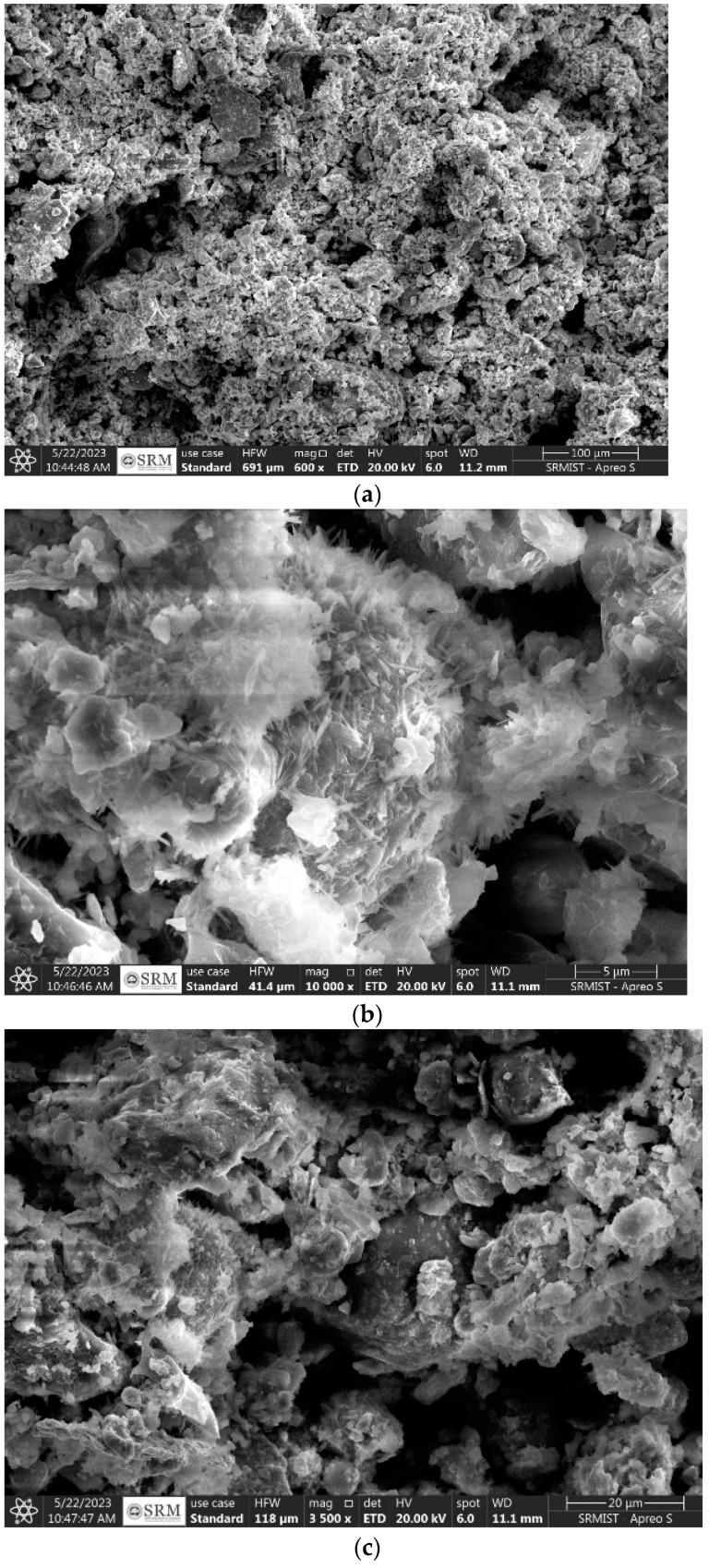
Microstructure analysis of iPyroxy; (**a**) iPyroxy micro structure at 100 nano microns; (**b**) iPyroxy microstructure at 5 nano microns; (**c**) iPyroxy micro structure at 20 nano microns.

Fool’s gold, or iron pyrite, is a sulfide mineral with the chemical formula FeS_2_, which is the result of the bonding of iron (Fe) with sulfur (S) [51,52]. Iron pyrite’s formation is intimately related to several geological events. It usually forms in igneous, metamorphic, and sedimentary settings. Iron pyrite may be found in organic-rich sediments and is often seen as nodules or concretions in sedimentary rocks. It may metamorphose in rocks exposed to high pressure and temperature, such as metamorphosed slates or shales. Iron pyrite may also be formed via igneous processes; the mineral precipitates from hydrothermal fluids connected to magmatic activity. Iron pyrite is also present in various rock types, such as disseminations and hydrothermal veins using Scanning Electron Microscopy, Hitachi High-Tech Corporation, Tokyo, Japan (Figure 1). The material has a cubic crystalline structure, striking brassy-yellow hue, and metallic sheen.

The composite material composed of iron pyrite and epoxy resin improved electromagnetic wave transmission properties, which may be ascribed to the particular chemical ingredients included in its composition (Table 2). The composite’s conductivity, or ability to move electrons efficiently, results from the carbon (C) content. By altering the material’s dielectric characteristics, oxygen (O) and fluorine (F) atoms help to lessen interference and signal loss. The structural stability and overall electrical conductivity of the composite are improved by the addition of silicon (Si), aluminum (Al), and magnesium (Mg). Sulfur (S) is added because it enhances charge transmission, further increasing conductivity. Calcium (Ca) acts as a moderator, affecting the dielectric constant and, in turn, wave transmission properties. Lastly, the iron (Fe) present in the iron pyrite component improves the composite’s overall magnetic characteristics. This combination of components reduces reflection and enhances electromagnetic wave transmission by maximizing the material’s impedance matching and promoting effective charge propagation. In addition to enabling a flexible platform for applications in electromagnetic devices and communication systems where improved wave transmission and low reflection are critical, the epoxy resin matrix offers structural support, guaranteeing the integrity of the composite.

## 3. Performance Analysis of iPyroxy Block

Iron pyrite, often referred to as fool’s gold (FeS_2_), is a mineral that may be extracted from sedimentary and metamorphic rock formations. Upon extraction, it is subjected to a crushing process to provide a fine powder with a particle size of around 300 microns. The crushing increases the surface area of the pyrite particles and facilitates a more uniform dispersion of the material when combined with a polymer matrix. Powdered iron pyrite may be efficiently integrated with commercially available epoxy resin for use in composite products. The epoxy resin acts as a resilient adhesive binder, creating a solid composite that leverages the distinctive characteristics of iron pyrite.

**Figure 2:**
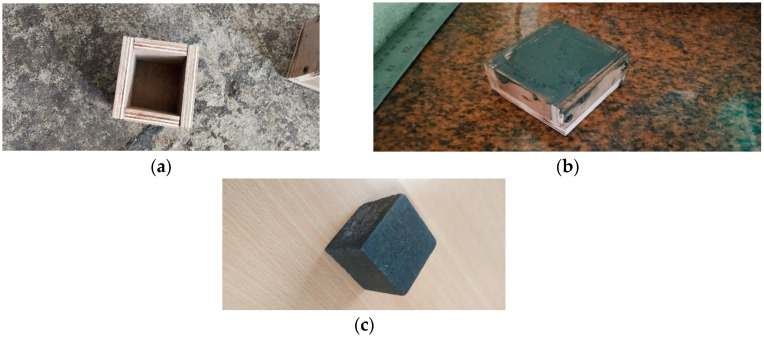
(**a**) Particular mold; (**b**) casting of 6iPyroxy block; (**c**) iPyroxy block.

iPyroxy blocks are formed by combining iron pyrite with epoxy resin in a ratio of 3:2, consisting of three parts iron pyrite to two parts epoxy resin. The liquid is thereafter put into a standard mold measuring 5 cm × 5 cm × 5 cm. After molding, the produced block undergoes a curing step lasting 24 h. The epoxy resin undergoes polymerization throughout this curing period, creating a robust matrix encapsulating the iron pyrite particles. The curing process is crucial for the iPyroxy block to attain optimal mechanical strength, durability, and structural integrity. The resulting composite material demonstrates mechanical properties derived from the cured epoxy resin matrix in conjunction with the electrical conductivity of iron pyrite. The 24 h curing period guarantees that the iPyroxy block is fully solidified, making it appropriate for use in various industries, including construction and electronics, or any other area that needs a composite material with specific mechanical and electrical properties (Figure 2).

iPyroxy’s remarkable dielectric value of 9.9 indicates good insulating qualities. This number indicates the material’s resistance to electrical current flow, making it useful as a dielectric in various settings. The higher dielectric constant of iPyroxy results from the 3:1 ratio of epoxy resin to iron pyrite (metallic brilliance). Because of its high dielectric value, iPyroxy may be used in processes that need electrical resistance and insulation, such as making capacitors, electronic components, or insulating materials.

### 3.1. Electromagnetic Wave Transmission Rate

Applying the following formula to a material with a dielectric constant ($\varepsilon_{r}$) of 9.8 will provide the electromagnetic wave transmission rate (*T*) for a 2.4 GHz signal. This means that the projected electromagnetic wave transmission rate for a 2.4 GHz signal is around 31.9% for a material having a dielectric constant of 9.8. This indicates that at a frequency of 2.4 GHz, around 31.9% of the electromagnetic waves pass through the material and depending upon other conditions, the remaining portion may reflect or absorb. Note that this is an oversimplified estimate and that the natural transmission qualities may vary depending on other parameters, including the characteristics of the material, behavior at different frequencies, and the particular circumstances surrounding the interaction of the electromagnetic wave with the material.

(1) $$\begin{array}{c} T=\frac{1}{\sqrt{\varepsilon_{r}}}\\ T=0.319 \end{array}$$

### 3.2. UV Absorption and Band Gap

**Figure 3:**
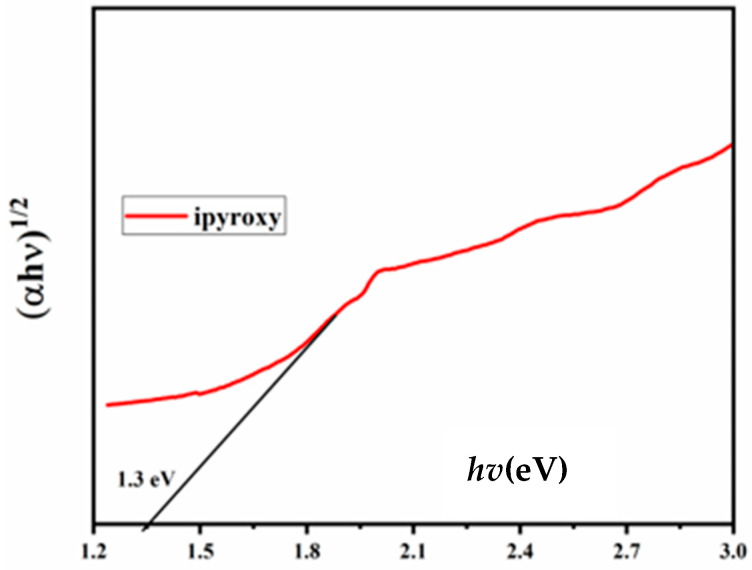
iPyroxy band gap.

The band gap and absorption properties of iPyroxy blocks may be efficiently examined by UV spectroscopy—an essential method for assessing the optical properties of materials. This technique entails subjecting iPyroxy materials to ultraviolet light and analyzing their absorption spectra. The initiation of UV absorption in the spectrum facilitates the band gap calculation, defined as the energy disparity between the conduction and valence bands. The observed band gap of iPyroxy is 1.3 eV, categorizing it as a semiconductor (Figure 3). The band gap denotes the energy necessary for electron transitions and indicates moderate electrical conductivity. The absorption peaks identified in UV spectroscopy elucidate the electronic alterations within the material, while distinct absorption values at different wavelengths reveal the interaction of UV light with the iPyroxy structure, enhancing comprehension of its electronic characteristics. Through UV spectroscopy, researchers can enhance the efficacy of iPyroxy for essential applications, including the creation of electronic devices and optoelectronic materials. The 1.3 eV band gap indicates advantageous optical properties within a defined frequency spectrum, rendering iPyroxy blocks appropriate for antenna substrates to improve data transmission. Moreover, their semiconductor characteristics make them suitable for optoelectronic devices, including LEDs and solar cells. The distinctive absorption properties of iPyroxy render it suitable for sensors, electrical components, and photonic applications, allowing effective light manipulation and detection. Comprehending these features is essential for developing technologies dependent on efficient light interaction.

### 3.3. Absorption Value

**Figure 4:**
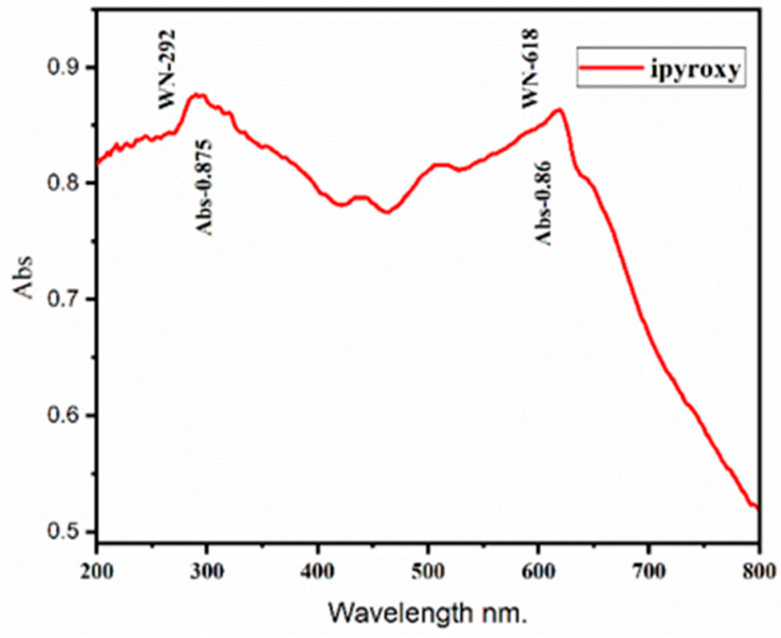
Absorption value of iPyroxy block.

The absorption characteristics of the iPyroxy block via UV-Vis spectroscopy is measured for the fabricated block. UV light was applied to the sample, and the resultant spectra were carefully analyzed to ascertain the material’s absorption characteristics. The iPyroxy block was produced using a precise 3:2 ratio of iron pyrite to epoxy resin, followed by a 24 h curing duration to guarantee its structural stability. The UV-Vis measurement indicated an absorption value of 0.875% for the iPyroxy block, correlating to a band gap energy of 1.3 eV (Figure 4). This discovery demonstrates the effective engagement with UV energy, establishing the material as appropriate for applications requiring selective absorption and emission of electromagnetic radiation. The absorption value of 0.875% indicates the material’s moderate absorption in the UV spectrum, facilitating its use in optoelectronics where these properties enhance energy transfer and signal transmission. The UV spectroscopic analysis of the iPyroxy block reveals a somewhat sluggish absorption of UV light within the examined spectrum, as seen by the documented absorption value.

### 3.4. Antenna Specification

A rectangular microstrip antenna operating from 1 to 15 GHz was designed using an FR4 substrate with a thickness of 1.6 mm, targeting dual-band operation at 4 GHz and 13.7 GHz. The antenna is impedance-matched to 50 ohms to ensure optimal power transfer and minimize reflection losses. The substrate’s dielectric constant (*ε_r_* = 4.4) and thickness influence the antenna’s bandwidth and radiation characteristics. The dual-band operation allows applications in the C-band (4 GHz), typically used in satellite communication and radars, and the Ku-band (13.7 GHz), suitable for high-speed data transmission and satellite broadcasting. Critical design challenges include maintaining efficiency and minimizing losses at higher frequencies due to the properties of FR4 as well as precise fabrication to achieve accurate resonances at both operating frequencies. The proposed antenna offers broadside radiation patterns and efficient performance across the designed frequency bands.

### 3.5. S Parameter Using Vector Network Analyser

The iPyroxy block within a specific frequency range may be thoroughly understood by examining its S-parameters (Scattering Parameters) [47]. Beginning at 1.000 GHz, the study is conducted up to 15.000 GHz, which is the breaking point. The amplitudes and phases of incident and reflected signals at various network ports are related by complex coefficients called S-parameters. In this instance, reference plane 1 is set 3.276 cm from the origin, whereas reference plane 2 is 3.215 cm away. To correctly depict the electrical properties of the iPyroxy block throughout the designated frequency spectrum, these reference planes, which identify the locations at which the S-parameters are measured, are necessary. The block’s signal integrity, transmission, and reflection characteristics will be better understood by examining the S-parameter data collected at the designated reference planes and throughout this frequency range.

### 3.6. S11 Reflection Coefficient

Measuring the reflected signal when an electromagnetic wave strikes a material is necessary to analyze the S11 parameter for the iPyroxy block. A high S11 value indicates significant reflection and possible signal loss, while less reflection and a better impedance match are indicated by a low S11 value.

### 3.7. S11 Reflection Coefficient with iPyroxy Block

**Figure 5:**
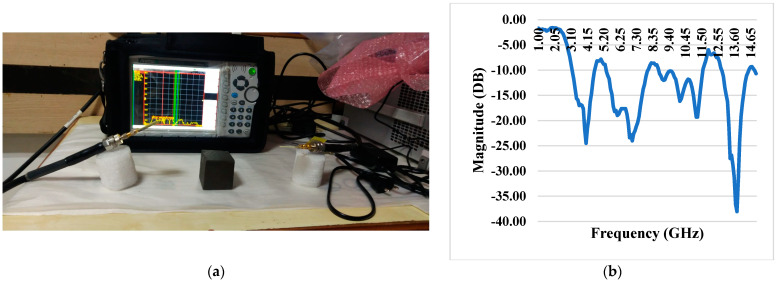
(**a**) S11VNA with iPyroxy block; (**b**) reflection coefficient with iPyroxy block.

The composite material has remarkable reflective performance, especially regarding electromagnetic waves, as shown by a constant low reflection coefficient over the whole operating frequency range. When an iPyroxy block is included in the composite, the S11 reflection coefficient is tested, and the results are consistently well below −10 dB (Figure 5). This indicates a low amount of energy lost due to reflection and an effective decrease in the size of reflected signals. By absorbing incoming electromagnetic waves and matching their impedance, the iPyroxy block, likely a mixture of iron pyrite and epoxy resin, helps to reduce the reflection coefficient. Because of its low reflection coefficient, this composite material is well-suited for various communication systems, radar technologies, and other electromagnetic devices that require optimal performance over a wide frequency spectrum. These applications place a high value on signal integrity and minimal signal loss.

### 3.8. S11 Reflection Coefficient Without iPyroxy Block

**Figure 6:**
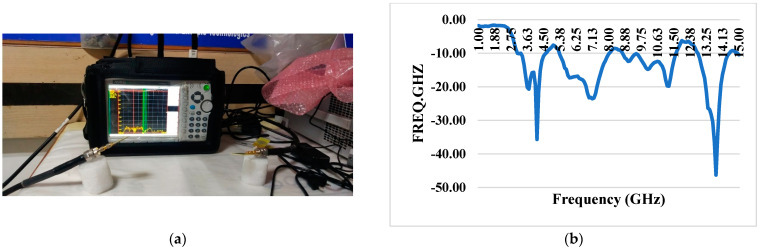
(**a**) S11 VNA without iPyroxy block; (**b**) S11 Reflection coefficient without iPyroxy block.

The material’s reflective properties are noteworthy, as evidenced by a consistently low reflection coefficient that remains well below −10 dB throughout the operational frequency range. The S11 reflection coefficient, measured without incorporating an iPyroxy block in the composite, demonstrates the material’s inherent ability to minimize signal reflection and energy loss (Figure 6). This suggests that the composite, likely comprised of elements such as epoxy resin, lacks significant impedance mismatches that would otherwise lead to signal reflection. The achievement of a reflection coefficient below −10 dB without the iPyroxy block underscores the material’s intrinsic characteristics, making it suitable for applications where signal integrity and minimal energy loss are critical considerations. This performance trait enhances the material’s viability in various electromagnetic applications, including antennas, wireless communication systems, and other devices where efficient signal transmission is essential across a diverse frequency range.

### 3.9. S11 Reflection Coefficient With and Without iPyroxy Block

**Figure 7:**
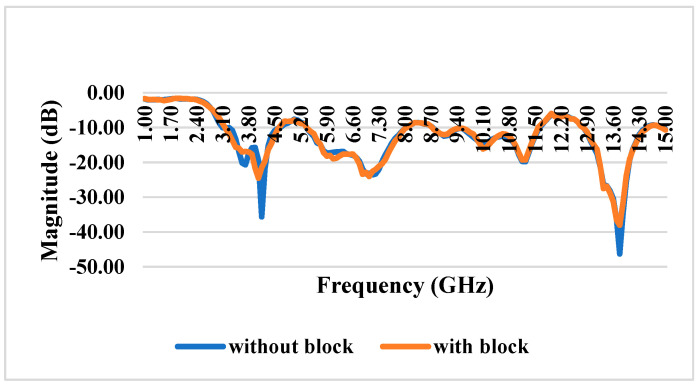
S11 Reflection coefficients with and without iPyroxy block.

The reflection coefficient measurements for the iPyroxy block, presented in Figure 7, were conducted using a Vector Network Analyzer (VNA) over a frequency range of 1 GHz to 15 GHz. The iPyroxy block was fabricated by mixing iron pyrite (FeS_2_) and epoxy resin in a 3:2 weight ratio, followed by a curing process of 24 h to ensure structural integrity and stable dielectric properties. The S11 reflection coefficients were measured with and without the iPyroxy block to assess its impact on signal reflection. The results indicate that with the iPyroxy block, the reflection coefficient remained consistently below −10 dB across the entire frequency range, demonstrating effective impedance matching and minimal signal reflection. In contrast, the measurements taken without the block also stayed below −10 dB, but including the iPyroxy block notably enhanced reflection performance at specific frequencies. These findings illustrate the ability of the iPyroxy block to reduce reflection losses and improve impedance matching, making it particularly suitable for applications in antennas and radar systems, where low reflectivity and high signal integrity are crucial. The manuscript has been revised to incorporate these experimental details and results.

## 4. S21 Transmission Coefficient

The transmitted signal is measured when an electromagnetic wave interacts with the material in the iPyroxy block to analyze the S21 parameter. Considerably more signal attenuation is suggested by a low S21 value than a high one, indicating inefficient transmission and little signal loss.

### 4.1. S21 Transmission Coefficient With and Without Block

**Figure 8:**
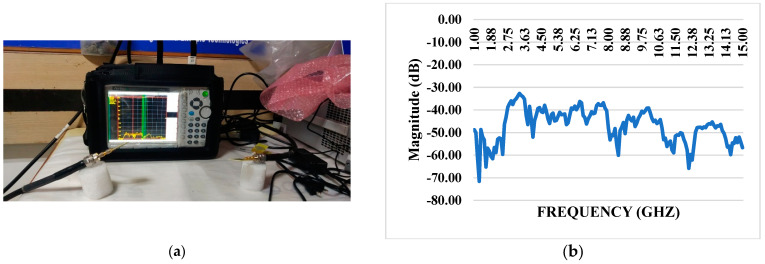
(**a**) S21 VNA without iPyroxy block; (**b**) S21 transmission coefficient without block.

The system’s mutual coupling coefficient is quite good; it constantly measures well below −15 dB across the operational range. The S21 transmission coefficient, measured without a blocking device, indicates the system’s intrinsic capacity to preserve low coupling among its constituents. Its low mutual coupling coefficient highlights the system’s ability to convey signals efficiently without significant interference or energy transfer between coupled units (Figure 8). The S21 transmission coefficient performance, especially without a blocking device, demonstrates the system’s intrinsic isolation and decoupling properties. This system is well-suited for use in wireless communication systems, phased-array antennas, and other scenarios where precise signal transmission and reception are crucial because of these attributes, which are critical in applications where signal integrity and isolation between different components or channels are paramount.

### 4.2. S21 Transmission Coefficient with Block

**Figure 9:**
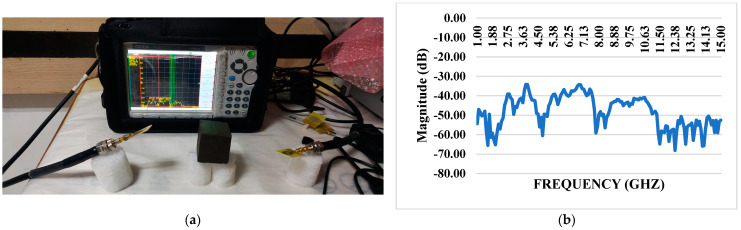
(**a**) S21 VNA with iPyroxy block; (**b**) S21 transmission coefficient without block.

The system demonstrates exceptional performance with a mutual coupling coefficient consistently measuring below −15 dB across the entire operating range. The S21 transmission coefficient, evaluated without including a blocking component, further emphasizes the system’s capability to transmit signals efficiently with minimal coupling between elements. The low mutual coupling coefficient signifies effective isolation between different components or channels within the system (Figure 9). The S21 transmission coefficient, unaffected by a blocking element, showcases the inherent ability of the system to maintain a high level of signal integrity and minimize signal leakage between interconnected elements. This performance characteristic is crucial in applications where precise signal transmission is vital, such as in wireless communication systems where minimizing interference between channels is essential for achieving reliable and high-quality communication.

### 4.3. S21 Transmission Coefficient With and Without Block

**Figure 10:**
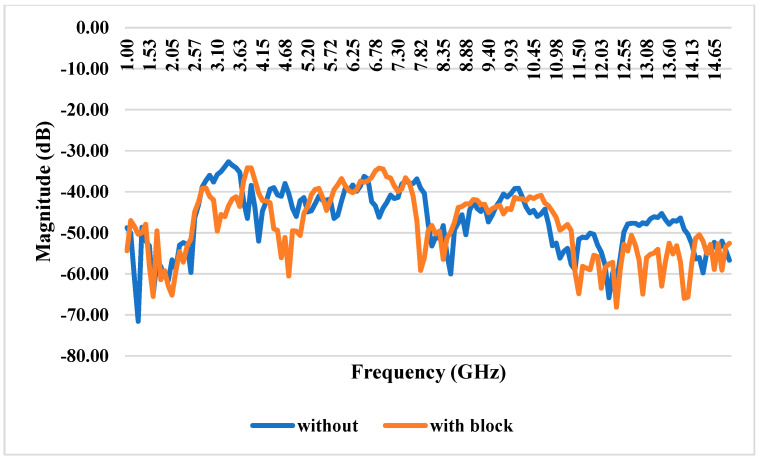
S21 transmission coefficient with and without block.

The structural characteristics of these materials are analyzed using transmission electron microscopy (TEM) to elucidate their shape and interface quality. The mutual coupling coefficient of the system, which signifies effective isolation between connected components, consistently registers values significantly below −15 dB across the entire operating spectrum. This is crucial for ensuring minimal interference among components. An examination of the S21 transmission coefficient without a blocking device shows that the system efficiently transmits signals with low coupling, as depicted in Figure 10. Incorporating an iPyroxy block results in minimal changes to the S21 transmission coefficient, indicating that the block has a negligible effect on the mutual coupling characteristics of the system. The blocking component maintains strong transmission capacity while simultaneously facilitating impedance matching and signal absorption, evidenced by a transmission rate of 90% when the iPyroxy block is included. These attributes make the system ideal for applications requiring reliable and high-quality communication across a wide operating range, such as phased-array antennas and wireless communication systems, where precise signal transmission and minimal coupling are critical. The S21 transmission coefficient for the system with and without the iPyroxy block is over a frequency range of 1 GHz to 15 GHz, highlighting the block’s impact on signal transmission between the input and output ports. Without the iPyroxy block, the S21 values exhibit relatively higher transmission levels, with minimal attenuation across the frequency spectrum. This suggests that the absence of the block allows for more excellent signal passage through the system, resulting in lower coupling losses.

Conversely, when the iPyroxy block is present, a notable drop in the S21 transmission coefficient occurs, especially at higher frequencies. This reduction implies that the iPyroxy block introduces additional impedance matching and signal absorption, decreasing transmission while enhancing selectivity. The interaction of the block with electromagnetic waves likely contributes to this variation by reducing mutual coupling and reflecting or absorbing a portion of the transmitted energy, which is advantageous for applications requiring signal isolation.

## 5. Simulation Analysis

A MATLAB 2020 simulation was conducted to evaluate the S21 transmission coefficient and S11 reflection coefficient for a rectangular microstrip antenna designed for dual-band functionality between 1 and 15 GHz. The antenna, fabricated using a FR4 substrate (*ε_r_* = 4.4) and with a thickness of 1.6 mm, is designed for frequencies of 4 GHz (C-band) and 13.7 GHz (Ku-band). The simulation assessed the S21 and S11 parameters in both the presence and absence of obstructions to analyze the antenna’s transmission and reflection characteristics. The S21 transmission coefficient without the block demonstrated superior transmission efficiency. However, the inclusion of the block resulted in considerable attenuation. The S11 reflection coefficient exhibited increased reflection with the introduction of the block, affecting overall impedance matching and efficiency. The experimental findings closely corresponded with the predicted data, confirming that the engineered antenna sustains efficient performance over the specified bands. Minor discrepancies between experimental and simulated values were observed due to manufacturing tolerances and material losses at high frequencies.

### 5.1. Simulation Results of S21 Transmission Coefficient Without Block

**Figure 11:**
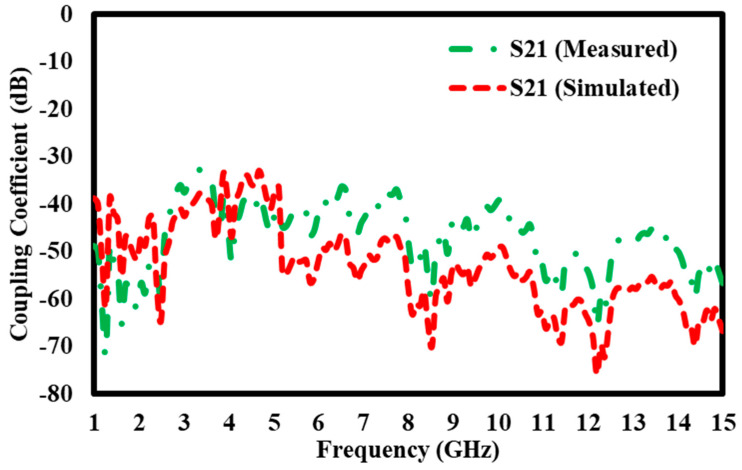
S21 transmission coefficient without block.

The graph compares the S21 coupling coefficients (in dB) measured and computed throughout a frequency spectrum from 1 GHz to 15 GHz. The Y-axis displays the coupling coefficient from 0 dB to −80 dB, reflecting energy transmission efficiency between the system’s two ports, whilst the X-axis denotes frequency in GHz. Two separate curves are illustrated: the green dashed line represents the measured S21 values acquired via empirical testing, while the red dashed line denotes the simulated S21 values generated by computational modelling. Both graphs demonstrate analogous broad patterns, with several peaks and troughs that indicate variations in signal transmission efficiency over the frequency spectrum. The curves show a significant level of agreement in the lower frequency range, namely between 2 GHz and 7 GHz, where the measured and simulated values nearly correspond (Figure 11), indicating dependable performance forecasts in this domain. However, significant disparities emerge once the frequency surpasses 7 GHz, causing the two curves to diverge in their behavior. This discrepancy indicates possible enhancements for the simulation model, emphasizing inconsistencies that may affect its precision in forecasting real-world performance, particularly at elevated frequencies. The graph underscores the significance of juxtaposing actual observed data with predicted outcomes, providing a basis for improving the modelling methodology to improve accuracy over the whole frequency range.

### 5.2. Simulation Results of S11 Reflection Coefficient for Without Block

**Figure 12:**
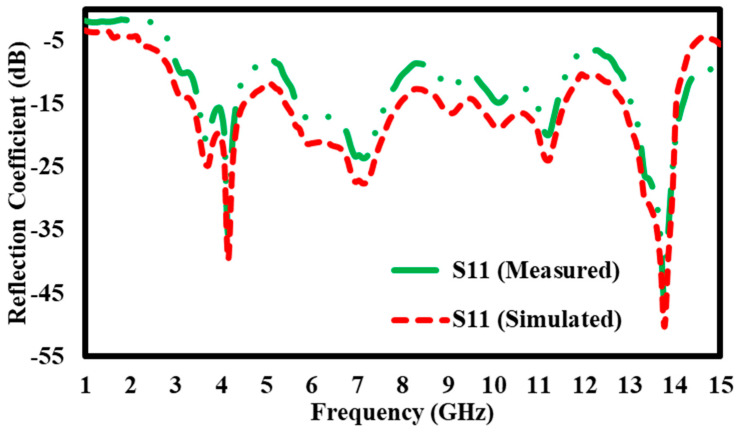
S11 Reflection coefficient without block.

The graph compares the observed and simulated S11 reflection coefficients (in dB) throughout a frequency range of 1 GHz to 15 GHz. The Y-axis denotes the reflection coefficient, from −5 dB to −55 dB, signifying the quantity of signal reflected from the device input. The X-axis represents the frequency in GHz. Two curves are depicted: the green dashed line indicates the measured S11 values, whilst the red dashed line denotes the simulated S11 values. Both curves show analogous tendencies, with identical peaks and troughs across the frequency spectrum, while some discrepancies between the measured and simulated data are apparent (Figure 12). The observed S11 exhibits pronounced dips, especially at 4 GHz and 14 GHz, where the reflection coefficient decreases markedly to about −45 dB. The simulated results show a similar general pattern but have less dramatic dips, suggesting somewhat stronger reflection in certain areas. Between 5 GHz and 12 GHz, the two curves exhibit a closer correlation, while discrepancies in magnitude remain at some intervals. The graph demonstrates a significant connection between observed and simulated S11 values, especially at crucial frequencies like 14 GHz, where both curves intersect strongly. The disparities at specific peaks and troughs indicate possible areas for enhancing the simulation model to reflect real-world performance accurately.

### 5.3. Simulation Results of S11 Reflection Coefficient for Block

**Figure 13:**
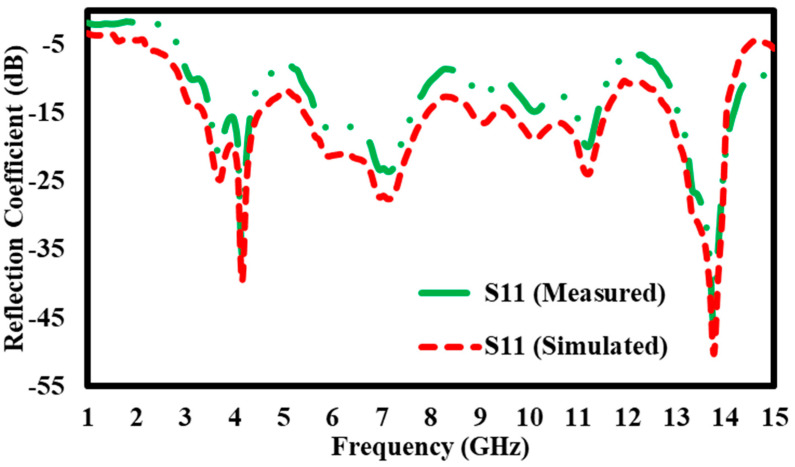
S11 Reflection coefficient for block.

The graph illustrates the S11 reflection coefficient for an antenna system with a block (Figure 13) spanning a frequency range of 1 GHz to 15 GHz. The S11 coefficient quantifies the power reflected to the source, acting as a vital metric for evaluating the efficacy of impedance matching, particularly in antenna or network configurations. The X-axis represents frequency in GHz, whilst the Y-axis illustrates the reflection coefficient in decibels (dB), with lower values indicating less signal reflection and enhanced impedance matching. The solid green line in the graph denotes the observed S11 values derived from empirical testing, while the dashed red line illustrates the simulated S11 values acquired from computational modelling. Both curves exhibit many troughs at distinct frequency locations, signifying diverse amounts of reflection over the frequency spectrum. A notable dip occurs at roughly 4 GHz when the measured and simulated S11 values decrease to about −40 dB, indicating near-optimal impedance matching at this frequency. Additional dips are seen at 5 GHz, 9 GHz, and 13 GHz, but the depth and precise frequency locations exhibit slight variations between the measured and simulated outcomes. These discrepancies presumably result from actual defects in the physical system or the intrinsic limits of the simulation model. Notwithstanding these slight discrepancies, the overarching pattern between the measured and simulated data is analogous, corroborating the model’s precision throughout most of the frequency spectrum. This research is crucial for assessing the performance of systems such as antennas because reducing the S11 reflection coefficient within specific frequency bands may markedly boost energy transmission and improve the system’s overall efficiency.

### 5.4. Simulation Results of S21 Transmission Coefficient with Block

**Figure 14:**
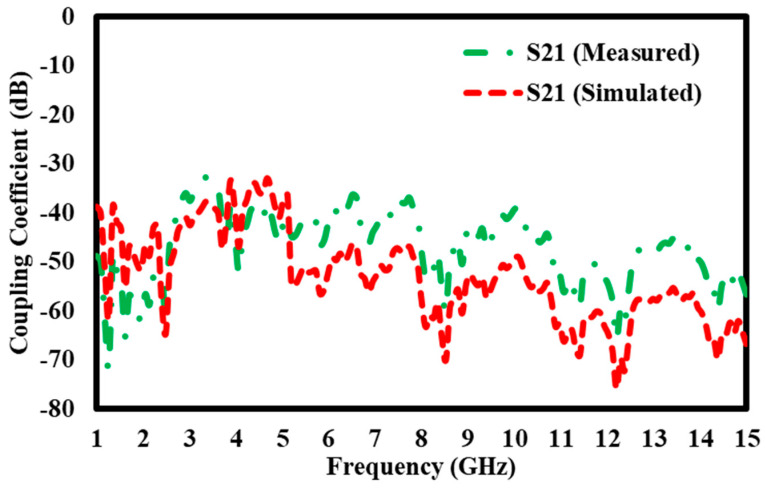
S21 transmission coefficient with block.

The graph depicts the S21 transmission coefficient for an antenna system with a block (Figure 14) spanning a frequency range of 1 GHz to 15 GHz. The S21 coefficient quantifies the extent of energy transfer between two ports in the system, with lower S21 values signifying more signal loss and higher S21 values indicating more effective energy transfer. The graph’s X-axis illustrates GHz frequency, while the Y-axis denotes the coupling coefficient in decibels (dB), spanning from −10 dB to −80 dB. The green dotted line represents the measured S21 values acquired from empirical testing, whereas the red dashed line illustrates the simulated S21 values gained from computational modelling. Both graphs exhibit similar tendencies across the frequency spectrum, while discrepancies in amplitude and particular frequency spots are noted. Between 2 GHz and 7 GHz, the S21 coupling coefficients range from −40 dB to −60 dB, indicating substantial energy transmission. At around 6 GHz, a significant peak is seen when the measured and simulated values align at roughly −40 dB, signifying optimum coupling efficiency within this range. Above 7 GHz, the results oscillate between −40 dB and −70 dB, indicating heightened unpredictability in energy transmission. The most significant disparities between the observed and simulated values are between 12 GHz and 14 GHz when the simulation indicates reduced coupling relative to the measured data. The discrepancies may arise from external sources, such as interference or system defects, which are not considered in the simulation model. The graph offers significant insights into the energy transfer efficiency of the antenna system with the block, emphasizing locations where design alterations might improve coupling performance throughout the frequency spectrum. The comparison of measured and simulated outcomes indicates possible areas for model refinement to enhance the accuracy of real-world performance predictions.

### 5.5. Simulation Results of S11 Reflection Coefficient With and Without 

**Figure 15:**
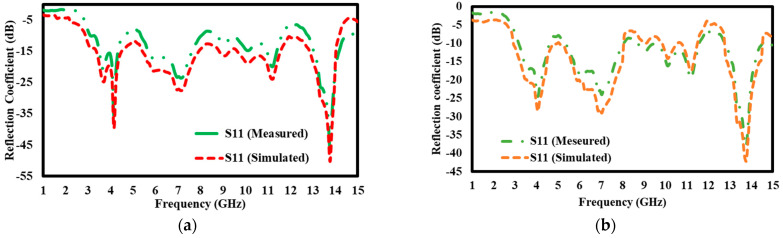
S11 Reflection coefficient (**a**) with a block; (**b**) without block.

The findings of the simulated study of the S11 reflection coefficient demonstrate a considerable technical difference in the performance of the antenna design with and without the block (Figure 15: (a) with the block and (b) without the block). The S11 parameter, measured in decibels (dB), measures the reflection of the signal input towards the source, with lower values indicating enhanced impedance matching and less signal reflection. In the scenario including the block, the antenna attains a reflection coefficient of −40 dB at 4 GHz, signifying little signal reflection. This indicates the antenna is almost optimally matched at this frequency, enabling efficient energy transmission with little loss. Additionally, the performance is enhanced further at an elevated frequency of 13.7 GHz, achieving a reflection coefficient of −50 dB. This deficient number signifies outstanding impedance matching, facilitating the transmission of almost all input power without reflection and enhancing the antenna’s performance at elevated frequencies.

In contrast, the antenna design lacking the block exhibits somewhat elevated reflection coefficients. At 4 GHz, the reflection coefficient measures −28 dB, indicating a significant increase in signal reflection relative to the design using the block. This suggests that impedance matching is less efficient in the absence of the block, resulting in increased signal reflection. At 13.7 GHz, the reflection coefficient enhances to −43 dB, surpassing the performance at 4 GHz, but it remains inferior to the comparable value with the block. This indicates that while the antenna devoid of the block has adequate performance, its efficiency is inferior to that of the block-integrated design, especially at elevated frequencies. Incorporating the block into the antenna design markedly improves impedance-matching properties, particularly at pivotal frequencies like 4 GHz and 13.7 GHz. The block’s presence reduces signal reflection, enhancing performance, especially at elevated frequencies where the reflection coefficient is lowered to levels as low as −50 dB. This modification emphasizes the block’s function for improving the antenna’s overall efficiency, making it an essential component in applications requiring high-frequency performance and low energy loss.

### 5.6. Simulation Results of S21 Transmission Coefficient With and Without Block

**Figure 16:**
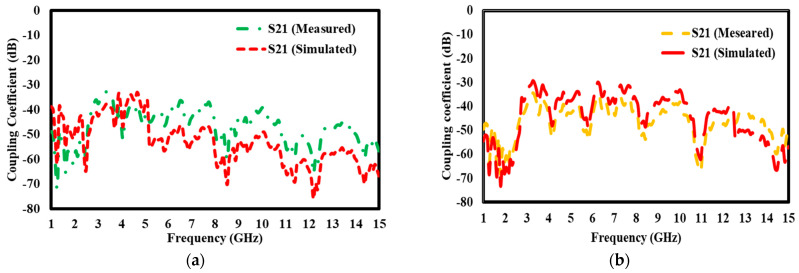
S21 transmission coefficient (**a**) with block; (**b**) without block.

The S21 transmission coefficient findings for both designs, with and without the block, illustrate the mutual coupling behavior between the antenna components spanning the frequency range of 1 GHz to 15 GHz (Figure 16: (a) with the block and (b) without the block). Mutual coupling denotes the electromagnetic interaction among antenna components, potentially resulting in interference and diminished performance. The mutual coupling coefficient continuously stays below −15 dB in all arrangements, indicating no electromagnetic interference among the antenna parts. A coupling value of −15 dB suggests that less than 3.16% of the power is transmitted between components, hence maintaining adequate isolation and mitigating substantial signal deterioration. The antenna with the block exhibits minimal mutual coupling, indicating that the block facilitates improved isolation and minimizes undesirable electromagnetic interactions among the components. This enhanced isolation guarantees that each antenna element functions autonomously, unaffected by signals from adjacent components, thereby enhancing the antenna’s performance across the frequency spectrum.

In the design without the block, the mutual coupling stays below −15 dB, indicating that the antenna retains enough isolation and little interference but somewhat elevated coupling values relative to the configuration with the block. In conclusion, both antenna topologies provide superior isolation with little mutual coupling over the frequency spectrum. The block’s presence further augments this isolation, reducing signal interference and enhancing overall performance, especially at elevated frequencies. The uniformity throughout the frequency spectrum guarantees the antenna’s efficient operation, making it appropriate for applications requiring little electromagnetic interference among components.

## 6. Application

The material’s superior transmission efficiency and minimal reflection losses make it ideal for antenna substrates, enhancing signal clarity and strength in wireless communication systems. It is also applicable in electromagnetic shielding, protecting sensitive electronic equipment from interference and improving overall device performance and reliability. Additionally, its configurable band gap optimizes sunlight absorption in solar cells, making it suitable for advanced photovoltaic systems. The material’s ability to effectively interact with electromagnetic waves enables its use in sensors for detecting various signals, supporting IoT and cutting-edge technological applications. Moreover, its high UV absorption capacity makes it valuable for creating UV sensors and devices for environmental monitoring and safety applications.

## 7. Conclusions

The iron pyrite-epoxy composite, formulated in a 3:2 weight ratio, uncovers significant insights into its electromagnetic wave properties. The composite has a controllable band gap of 1.3 eV, enabling selective absorption and emission of electromagnetic radiation, which is very beneficial for UV spectroscopy applications. The transmission coefficient recorded at a remarkable 90% throughout a frequency range of 1 GHz to 15 GHz, demonstrating exceptional transparency to electromagnetic waves and underscoring its potential for high signal integrity in sensing devices and communication systems. The composite has a low reflection coefficient of 10%, substantially reducing reflecting interference, which is essential for precise measurements in electromagnetic wave systems. The UV-Vis spectroscopy findings provide an absorption value of 0.875% at a band gap of 1.3 eV, highlighting the composite’s effective interaction with UV radiation. The S21 transmission coefficient varied from −10 dB to −80 dB, reaching a maximum of −40 dB at 6 GHz, signifying robust energy transfer characteristics appropriate for antenna applications. The S11 reflection coefficient ranged from −5 dB to −55 dB, exhibiting significant reductions between 4 GHz and 14 GHz, where it reduced to −45 dB, highlighting the composite’s efficacy in lowering signal reflection at essential frequencies. The advantages of iron pyrite include its natural abundance and inexpensive cost, making it an economically feasible choice for composite materials. The disadvantages pertain to its relatively poor stability under environmental conditions, potentially impacting its long-term application performance. These results highlight the iron pyrite-epoxy composite’s remarkable appropriateness for applications necessitating high transmission efficiency, little reflection, and proficient interaction with electromagnetic waves, especially as an antenna substrate.

## Figures and Tables

**Table 1:** Building materials’ electromagnetic wave absorption.

Materials Value	Electromagnetic Wave Absorption dB	References
Concrete	10–20	[40]
Brick	5–15	[41]
Gypsum board	8–25	[42]
Wood	5–12	[43]
Composite material	10–30	[44]
steel	20–40	[45]
Natural stone	20–40	[46]

**Table 2:** Chemical composition in iPyroxy.

Element	Net Counts	Weight %	Atom %	Atom % Error	Formula
C	131	8.21	16.10	±1.11	C
O	449	33.63	49.48	±2.87	O
F	17	1.49	1.84	±2.49	F
Mg	58	1.28	1.24	±0.23	Mg
Al	260	4.24	3.70	±0.24	Al
Si	495	7.15	5.99	±0.38	Si
S	93	1.10	0.81	±0.12	S
Ca	1079	16.62	9.76	±0.29	Ca
Fe	713	26.28	11.08	±0.71	Fe
Total		100.00	100.00		

## Data Availability

The original contributions presented in the study are included in the article, further inquiries can be directed to the corresponding authors.

## References

[1] Kwe N.B., Yadav V., Kumar M., Savilov S.V., Yahya M.Z.A., Singh S.K. (2024). A Comparative Study of Dielectric Substrate Materials Effects on the Performance of Microstrip Patch Antenna for 5G/6G Application. J. Mater. Sci. Mater. Electron..

[2] Kedze K.E., Wang H., Park Y.B., Park I. (2022). Substrate Dielectric Constant Effects on the Performances of a Metasurface-Based Circularly Polarized Microstrip Patch Antenna. Int. J. Antennas Propag..

[3] Abdel Halim A.S., Abdel-Salam Z., Abdel-Harith M., Hamdy O. (2024). Investigating the Effect of Changing the Substrate Material Analyzed by Laser-Induced Breakdown Spectroscopy on the Antenna Performance. Sci. Rep..

[4] Qin M., Zhang L., Wu H. (2022). Dielectric Loss Mechanism in Electromagnetic Wave Absorbing Materials. Adv. Sci..

[5] Tütüncü B., Kösem M. (2022). Substrate Analysis on the Design of Wide-Band Antenna for Sub-6 GHz 5G Communication. Wirel. Pers. Commun..

[6] Zhou Z., Wei J., Luo Y., Clark K.A., Sillekens E., Deakin C., Sohanpal R., Slavík R., Liu Z. (2023). Communications with Guaranteed Bandwidth and Low Latency Using Frequency-Referenced Multiplexing. Nat. Electron..

[7] Ali S.Z., Ahsan K., ul Khairi D., Alhalabi W., Anwar M.S. (2024). Advancements in FR4 Dielectric Analysis: Free Space Approach and Measurement Validation. PLoS ONE.

[8] Chaimool S., Prasert N., Rakluea C. Low-Cost FR-4 Metasurface-Enhanced Microstrip Patch Antenna Array for Wideband 5G Millimeter-Wave Applications. Proceedings of the 2024 IEEE International Workshop on Antenna Technology (iWAT 2024).

[9] Zahidul Islam M.D., Fu Y., Deb H., Khalid Hasan M.D., Dong Y., Shi S. (2023). Polymer-Based Low Dielectric Constant and Loss Materials for High-Speed Communication Network: Dielectric Constants and Challenges. Eur. Polym. J..

[10] Aleem A., Ghaffar A., Kiani N.M., Irshad M., Mehmood I., Shahzad M., Shahbaz A. (2021). Broad-Band Dielectric Properties of Teflon, Bakelite, and Air: Simulation and Experimental Study. Mater. Sci. Eng. B.

[11] Andrew J.J., Dhakal H.N. (2022). Sustainable Biobased Composites for Advanced Applications: Recent Trends and Future Opportunities—A Critical Review. Compos. Part. C Open Access.

[12] Samir A., Ashour F.H., Hakim A.A.A., Bassyouni M. (2022). Recent Advances in Biodegradable Polymers for Sustainable Applications. NPJ Mater. Degrad..

[13] Ayode Otitoju T., Ugochukwu Okoye P., Chen G., Li Y., Onyeka Okoye M., Li S. (2020). Advanced Ceramic Components: Materials, Fabrication, and Applications. J. Ind. Eng. Chem..

[14] Oses C., Toher C., Curtarolo S. (2020). High-Entropy Ceramics. Nat. Rev. Mater..

[15] Al-Oqla F.M., Omar A.A. (2015). An Expert-Based Model for Selecting the Most Suitable Substrate Material Type for Antenna Circuits. Int. J. Electron..

[16] Koziel S., Pietrenko-Dabrowska A. (2023). On Nature-Inspired Design Optimization of Antenna Structures Using Variable-Resolution EM Models. Sci. Rep..

[17] Xu Z., Hui J., Lv J., Wei D., Yan Z., Zhang H., Wang J. (2024). An Investigation of Methods to Enhance Adhesion of Conductive Layer and Dielectric Substrate for Additive Manufacturing of Electronics. Sci. Rep..

[18] De Guzman J.L.A., Guzman A.C.C.V. (2024). Design and Optimization of Micro-Strip Patch Antennas for Wireless Communication Systems—A Literature Review. Int. J. Res. Publ. Rev..

[19] Koziel S., Pietrenko-Dabrowska A. (2023). High-Efficacy Global Optimization of Antenna Structures by Means of Simplex-Based Predictors. Sci. Rep..

[20] Marasco I., Niro G., De Marzo G., Rizzi F., D’orazio A., Grande M., De Vittorio M. (2023). Design and Fabrication of a Plastic-Free Antenna on a Sustainable Chitosan Substrate. IEEE Electron. Device Lett..

[21] Aileen A., Suwardi A.D., Prawiranata F. (2021). Wi-Fi Signal Strength Degradation over Different Building Materials. Eng. Math. Comput. Sci. J..

[22] Suherman S. (2018). Wifi-Friendly Building to Enable Wi-Fi Signal Indoor. Bull. Electr. Eng. Inform..

[23] Dambal V.A., Mohadikar S., Kumbhar A., Guvenc I. Improving LoRa Signal Coverage in Urban and Sub-Urban Environments with UAVs. Proceedings of the 2019 International Workshop on Antenna Technology iWAT.

[24] Di Giacomo R., Neitzert H.C., Vertuccio L., Sorrentino A., Sabbatino S. (2011). Lecture Notes in Electrical Engineering: Foreword. Lect. Notes Electr. Eng..

[25] Wang B., Wei J., Yang Y., Wang T., Li F. (2011). Investigation on Peak Frequency of the Microwave Absorption for Carbonyl Iron/Epoxy Resin Composite. J. Magn. Magn. Mater..

[26] Jiang Y., Liu L., Yan J., Wu Z. (2024). Room-to-low temperature thermo-mechanical behavior and corresponding constitutive model of liquid oxygen compatible epoxy composites. Compos. Sci. Technol..

[27] Xu Y., Li Y., Hua W., Zhang A., Bao J. (2016). Lightweight Silver Plating Foam and Carbon Nanotube Hybridized Epoxy Composite Foams with Exceptional Conductivity and Electromagnetic Shielding Property. ACS Appl. Mater. Interfaces.

[28] Chen Y., Li C., Yang X. (2023). Simultaneous measurement of trace dimethyl methyl phosphate and temperature using all fiber Michaelson interferometer cascaded FBG. Opt. Express.

[29] Gao X., Yang W., Cheng L., Ding Y., Zhan J., Tan J. (2021). Epoxy Resin Composite Containing Nanocarbon-Coated Glass Fiber and Cloth for Electromagnetic Interference Shielding. J. Mater. Res. Technol..

[30] Cui M., Xiong S., Yang N., Wang Y., Wang Z., Luo M., Deguchi Y. (2024). Applications of laser-induced breakdown spectroscopy in industrial measurement and monitoring: Multi-technology combination. Appl. Spectrosc. Rev..

[31] Bian W., Yao T., Chen M., Zhang C., Shao T., Yang Y. (2018). The Synergistic Effects of the Micro-BN and Nano-Al_2_O_3_ in Micro-Nano Composites on Enhancing the Thermal Conductivity for Insulating Epoxy Resin. Compos. Sci. Technol..

[32] Yang Y., Zhang Z., Zhou Y., Wang C., Zhu H. (2023). Design of a Simultaneous Information and Power Transfer System Based on a Modulating Feature of Magnetron. IEEE Trans. Microw. Theory Tech..

[33] Wang J., Jiao J., Sun G., Yuan K., Guan Z., Wei X. (2019). Preparation and Microwave Absorption Performance of a Flexible Fe3O4/Nanocarbon Hybrid Buckypaper and Its Application in Composite Materials. RSC Adv..

[34] Zha S., Qu Z., Zhang J., Zheng D., Liu P. (2024). A Gain-Reconfigurable Reflector Antenna with Surface-Mounted Field-Induced Artificial Magnetic Conductor for Adaptive HIRF Prevention. IEEE Trans. Antennas Propag..

[35] Faruque M.R., Islam M.T., Islam S.S. (2018). Space Science and Communication for Sustainability.

[36] Zhang W., Kang S., Liu X., Lin B., Huang Y. (2023). Experimental study of a composite beam externally bonded with a carbon fiber-reinforced plastic plate. J. Build. Eng..

[37] Lyubutin I.S., Starchikov S.S., Lin C.R., Lu S.Z., Shaikh M.O., Funtov K.O., Dmitrieva T.V., Ovchinnikov S.G., Edelman I.S., Ivantsov R. (2013). Magnetic, Structural, and Electronic Properties of Iron Sulfide Fe 3S4 Nanoparticles Synthesized by the Polyol Mediated Process. J. Nanoparticle Res..

[38] Zhang C., Khorshidi H., Najafi E., Ghasemi M. (2023). Fresh, mechanical and microstructural properties of alkali-activated composites incorporating nanomaterials: A comprehensive review. J. Clean. Prod..

[39] Qin H., Jia J., Lin L., Ni H., Wang M., Meng L. (2018). Pyrite FeS_2_ Nanostructures: Synthesis, Properties and Applications. Mater. Sci. Eng. B.

[40] Buyukozturk O. (1997). Electromagnetic Properties of Concrete and Their Significance in Nondestructive Testing. Transp. Res. Rec..

[41] Xie S., Ji Z., Zhu L., Zhang J., Cao Y., Chen J., Liu R., Wang J. (2020). Recent Progress in Electromagnetic Wave Absorption Building Materials. J. Build. Eng..

[42] Liu T.T., Cao M.Q., Fang Y.S., Zhu Y.H., Cao M.S. (2022). Green Building Materials Lit Up by Electromagnetic Absorption Function: A Review. J. Mater. Sci. Technol..

[43] Zhou M., Gu W., Wang G., Zheng J., Pei C., Fan F., Ji G. (2020). Sustainable Wood-Based Composites for Microwave Absorption and Electromagnetic Interference Shielding. J. Mater. Chem. A.

[44] Herron D. (2004). Electromagnetic Wave Absorbing Materials.

[45] Richalot E., Bonilla M., Wong M.F., Fouad-Hanna V., Baudrand H., Wiart J. (2000). Electromagnetic Propagation into Reinforced-Concrete Walls. IEEE Trans. Microw. Theory Tech..

[46] Wei M., Song D., He X., Li Z., Qiu L., Lou Q. (2020). Effect of Rock Properties on Electromagnetic Radiation Characteristics Generated by Rock Fracture During Uniaxial Compression. Rock. Mech. Rock. Eng..

[47] Dos Santos E.C., Lourenço M.P., Pettersson L.G.M., Duarte H.A. (2017). Stability, Structure, and Electronic Properties of the Pyrite/Arsenopyrite Solid-Solid Interface-A DFT Study. J. Phys. Chem. C.

[48] Lou J., Bhobe A., Shu Y., Yu J. Analytical Calculation of Transformer Parameters by S-Parameters. Proceedings of the 2018 IEEE International Symposium on Electromagnetic Compatibility and 2018 IEEE Asia-Pacific Symposium on Electromagnetic Compatibility (EMC/APEMC).

[49] Kaur K., Malhotra S. (2017). Analyzing the Impedance of Reflection Coefficient of S-Parameters S11 and S22 Using Vector Network Analyzer & Recognization of Hand Gesture Using DTW. JETIR.

[50] Yang C., Wang J., Yang C. (2021). Estimation Methods to Extract Complex Permittivity from Transmission Coefficient in the Terahertz Band. Opt. Quantum Electron..

[51] Ozturk T., Hudlička M., Uluer İ. (2017). Development of Measurement and Extraction Technique of Complex Permittivity Using Transmission Parameter S 21 for Millimeter Wave Frequencies. J. Infrared Millim. Terahertz Waves.

[52] Fezai N., Ben Amor A. Measure of Reflection Factor S11 High Frequency. Proceedings of the International Conference on Advanced Systems and Electric Technologies (IC_ASET 2017).

